# Exercise-related T-wave oversensing: an underestimated cause of reduced exercise capacity in a pacemaker-dependent patient—a case report and review of the literature

**DOI:** 10.1007/s10840-019-00698-6

**Published:** 2020-01-23

**Authors:** Albert Topf, Lukas J. Motloch, Johannes Kraus, Franz Danmayr, Moritz Mirna, Christiane Schernthaner, Uta C. Hoppe, Bernhard Strohmer

**Affiliations:** Department of Cardiology, Clinic of Internal Medicine II, Paracelsus Medical University of Salzburg, Salzburg, Austria

**Keywords:** T wave oversensing, Pacemaker

## Abstract

A 62-year-old pacemaker-dependent patient presented to our department with a sudden onset of reduced physical capacity. While initial physical and pacemaker evaluations remained without specific findings, Holter-ECG monitoring revealed an abnormal rate response with unusual pauses during physical exercise. Consequently, closer evaluation of the pacemaker system revealed intermittent, exercise-related T-wave oversensing (TWOS). While TWOS remains a significant burden in ICD-patients, it might be an underestimated but clinically significant event in pacemaker patients. Further studies should evaluate the impact of TWOS in this patient population.

## Introduction

T-wave oversensing (TWOS) is an often-reported problem in ICD patients. It is a feared complication, which might lead to inappropriate shocks with a significant impact on the patient’s quality of life [1, 2]. Consequently, episodes of TWOS have significant impact on morbidity and mortality in ICD-patients. Although TWOS is expected to occur less frequently in pacemaker patients due to the fixed programming of the sensitivity threshold, little is known about the frequency and clinical impact of this unusual phenomenon. We present a case of a 62-year-old pacemaker-dependent patient, who presented to our department due to intermittent exercise-related TWOS.

## Case report

A 62-year-old pacemaker-dependent patient presented with an onset of reduced exercise capacity. In 2002, the patient underwent radiofrequency catheter ablation of persistent atrial fibrillation at another institution without lasting success. For prevention of tachymyopathy related to rapid atrial fibrillation, catheter ablation of the AV node was performed followed by implantation of a single chamber ventricular pacemaker (Table 1). Despite the initial improvement, the patient developed mild cardiomyopathy over the following years presumably due to the right ventricular apical pacing-induced left ventricular dyssynchrony. In 2017, he underwent catheter ablation of frequent ventricular bigeminy arising from the left ventricular outflow tract with a significant reduction of the ectopy during follow-up. During one of the last pacemaker visits, the percentage of ventricular stimulation was 97% and the ventricular sensitivity was adjusted from the nominal value of 3 to 1 mV in order to ensure sensing of the sporadic ectopic beats (Table 1). Later on, the patient complained about a reduced exercise capacity with significant dyspnea on mild exertion. Thus, he presented to our department for further investigation of his unclear deterioration.

**Table 1:** Programming of the single chamber pacemaker

Aggregate Lead	Biotronik Epyra SR Vitatron-Crystalline, bipolar
Mode/Rate	VVI CLS 65–120 ppm
Increase in frequency	2
Drop in frequency	0.5
Sensitivity (programmed)	1 mV
Sensitivity (nominal)	3 mV
Threshold	1.3 V @ 0.4 ms
Output programmed	2.4 V @ 0.4 ms
ERI	8.9 years

**Fig. 1:**
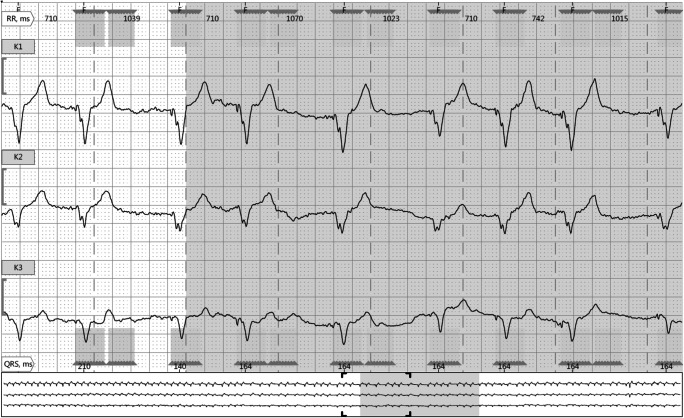
Holter ECG recording (at 25 mm/s) showed paced beats with irregular RR intervals ranging from 710 to 1040 ms with start of physical activity.

**Fig. 2:**
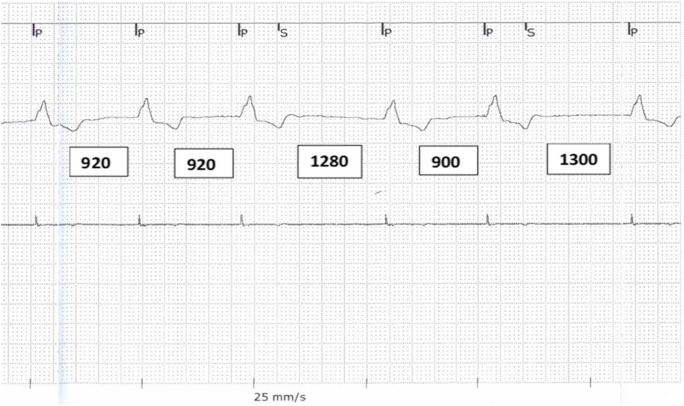
Pacemaker real-time ECG/EGM recording reveals intermittent exercise-related TWOS (S, sensing of T wave every second beat) while performing knee bends (paper speed 25 mm/s; marker channel on top, surface ECG, intracardiac bipolar EGM on bottom)

The clinical examination including transthoracic echocardiography and laboratory testing was unremarkable. The pacemaker interrogation at rest did not show any obvious abnormalities of the permanently implanted system. For further elucidation of the transient problem, a Holter-ECG was initiated. The continuous ECG monitoring revealed an inadequate elevation of the paced heart rate during exercise. Interestingly, these episodes showed repetitive pauses of 1.0–1.3 s in duration (Fig. 1). Therefore, a detailed pacemaker check was carried out including exercise provocation tests with real-time monitoring of the surface ECG and intracardiac EGM channels. The patient was prompted to perform knee bends for acceleration of the CLS sensor–driven pacing rate. During exercise, intermittent TWOS of some of the paced beats was noted, which provoked episodes of repetitive pauses with similar duration observed on the Holter-ECG recording (Figs. 1 and 2). All together 10 episodes of TWOS manifestations with inadequate pauses with a duration longer than the lower rate interval (LRI) were documented. This phenomenon of functional TWOS during exercise prevented an adequate increase of heart rate resulting in a reduced functional capacity of this pacemaker-dependent patient. At worst, the effective heart rate decreased to 70% of the LRI. To solve the problem, the sensitivity was decreased to the nominal value of 3 mV, which eliminated the exercise-related TWOS completely. After reprogramming, the patient’s capacity improved again and an appropriate rate response up to 120 ppm was confirmed with bicycle ergometry. The following Holter ECG monitoring documented an adequate increase in heart rate with absence of any suspicious pauses. As the patient remained asymptomatic for the following 6 months, the upgrade to a biventricular pacing device was postponed.

## Discussion

To the best of our knowledge, this is the first case report that describes a clinically relevant, symptomatic exercise-related TWOS in a pacemaker-dependent patient. TWOS is a well-known problem in ICD patients and may result frequently in inappropriate shocks. Because of these dramatic symptoms, algorithms were designed to prevent TWOS in this population [1–3]. However, in pacemaker patients, TWOS of intrinsic or paced beats is considered to remain clinically insignificant. Of note, only a few studies are dealing with this problem in pacemaker patients (Table 2). These investigations revealed a prevalence of TWOS between 1 and 9%. Although these episodes provoked bradycardia and false-positive EGM classification, no clinically relevant symptoms have been reported so far (Table 2). Consequently, algorithms to prevent these events are absent.

**Table 2:** Review of the literature dealing with TWOS

Authors	*N*	*N* with TWOS	Manifestation of TWOS	Reason for TWOS	Symptoms caused by TWOS
Michal Chudzik et al.	100	9	Pauses 1.1–1.6 s, temporary bradycardia in a 24 h ECG	A short programmed ventricular refractory period	-
S. Paraskevaidis et al.	362	2	False-positive episodes of VTs and nsVTs in the EGM storage	High programmed atrial sensitivity	-
Okreglicki et al (case report)	1	1	Bradycardia on ECG monitoring after surgery	High programmed ventricular sensitivity in a VVI pacemaker	-
Bohm et al. (case report)	1	1	Bradycardia on ECG at the family doctor	High programmed sensitivity in a VVI pacemaker	-

TWOS is more frequently associated with pathologies having an unfavorable R and T wave ratio including Brugada syndrome, hypertrophic cardiomyopathy, cardiac sarcoidosis, long QT syndrome, ischemic cardiovascular events, and high potassium levels [4]. Loss of CRT due to TWOS has been described in isolated case reports [5]. It has been reported previously that the T wave gets higher and tighter during exercise [6]. Consequently, the R/T wave ratio is changed in favor of TWOS. Additionally, a shortening of the PVARP and VRP under exercise accelerates the risk of TWOS. Unfortunately, the slew rate as a morphological discriminator is not helpful to sort out T waves from R waves.

According to the literature, some drugs including sacubutril/valsartan were reported to trigger TWOS [7]. In our case, the patient’s medication was not modified and therefore this reason seems to be unlikely. In the present case, the problem occurred after increasing the ventricular sensitivity to detect the underlying slow escape rhythm and ventricular extrasystoles. While after reprogramming the sensitivity TWOS oversensing was not present at rest, it occurred during physical exertion, most likely because of exercise-related electrical alterations including shortening of the ventricular refractory period and dynamic increase of the T wave.

From a technical point of view, there is no relationship reported between T wave oversensing and the closed loop stimulation (CLS) sensor system. The CLS sensor measures the intracardiac impedance at the tip of the electrode at rest and at exertion in the systole of the cardiac cycle. The difference between the impedance at rest and at exertion results in an adjustment of the frequency. The CLS sensor works completely independent from the intracardiac electrogram signals and is not influenced by the T wave amplitude [8].

Our observation might be limited by the fact that AVN ablation with consequent pacemaker implantation is nowadays rarely performed as therapy of refractory atrial fibrillation. However, the clinical phenomenon may occur also among the pacemaker-dependent population, such as in patients with intrinsic high-grade AV block.

Notably, TWOS was observed primarily on Holter-ECG recording with unexplained irregularities of pacing during physical activity. Thereafter, exercise provocation tests with real-time ECG/EGM monitoring revealed the underlying problem.

The present case underlines the clinical significance of both diagnostic tests in unclear pacemaker malfunction situations beyond the basic pacemaker interrogation at resting state.

Exercise-related TWOS was facilitated by an elevated sensitivity that was programmed to assure consistent sensing of sporadic ventricular ectopy. However, in pacemaker-dependent patients programming of high sensitivity should be carried out with caution, as oversensing of any type of signals and myopotentials may result in loss of stimulation. In clinical practice, this dynamic phenomenon may be under-recognized in the pacemaker population. Further studies are needed to investigate the clinical significance of exercise-related TWOS systematically.

## Conclusion

While TWOS remains a significant burden in ICD-patients, it may be an underestimated but clinically significant phenomenon in patients who need consistent ventricular pacing. The present case demonstrates the detrimental functional sequelae of dynamic exercise-induced TWOS in a patient who is dependent on adequate rate response after AV node ablation. Beyond basic pacemaker interrogation, meticulous tests during physical activity are necessary to unravel this transient malfunction in the individual patient.
